# The Interrelationship of Loneliness, Smartphone Addiction, Sleep Quality, and Students’ Attention in English as a Foreign Language Class

**DOI:** 10.3390/ijerph20043460

**Published:** 2023-02-16

**Authors:** Po-Chi Kao

**Affiliations:** Center for General Education, Chang Gung University, Taoyuan City 333, Taiwan; mk@mail.cgu.edu.tw

**Keywords:** loneliness, smartphone addiction, sleep quality, attention in EFL class

## Abstract

In this study, a research model comprising four variables (loneliness, smartphone addiction, sleep quality, and students’ attention in English as a foreign language class) was proposed and statistically examined. Previous literature has appeared to neglect these variables, which are considered to be essential to understanding students’ attention in EFL (English as a foreign language) class among college students. A total of 587 undergraduate students were recruited from a university in Taiwan to participate in the present study. The technique of structural equation modeling was adopted to test the hypotheses in the conceptual model. The findings of this study are: (1) smartphone addiction has a significant negative impact on students’ attention in EFL class; (2) smartphone addiction has a significant negative impact on sleep quality; (3) sleep quality has a significant positive impact on students’ attention in EFL class; (4) sleep quality partially mediates the relationship between smartphone addiction and students’ attention in EFL class; (5) loneliness has a significant positive effect on smartphone addiction. The results can enrich the present literature in the psychology of attention and mobile technology by providing an insight into the dynamics of these four variables.

## 1. Introduction

Technology has a significant influence on our lives; it has brought us both conveniences and challenges that we could not have imagined. In particular, multifunctional smartphones, despite being only little gadgets in size, have literally put the world at our fingerprints. Though smartphones have been used as educational tools, they can also distract students’ learning when they are used to play games, send messages, or social network with friends during class. Researchers [1,2] assert that the existence of smartphones has reduced students’ attention in class. In spite of the potential negative impact that smartphones may have on students’ attention in class, the devices have become a necessity that many university students cannot do without.

The motivation behind this research project was to untangle the dynamics of smartphone addiction and college students’ attention in EFL (English as a foreign language) class. Previous studies have mainly focused on how smartphone usage can create distractions, which in turn diminishes classroom learning and attention. The present study draws findings from previous literature and includes two new variables—loneliness and sleep quality. Apart from analyzing the impact of smartphone addiction on attention in EFL classroom settings, the impact of smartphone addiction on the quality of sleep was examined. The impact of loneliness on smartphone addiction was also tested in this study. Finally, the researcher looked into whether a mediating effect of sleep quality existed between smartphone addiction and attention in EFL class.

### 1.1. Rationale of the Research

A scarcity of research on the impact of loneliness on smartphone addiction, as well as smartphone addiction’s effect on the quality of sleep and attention in EFL class among the university student population, has prompted this study. There have been studies investigating the extra cognitive load driven by multitasking and its impact on students’ understanding of learning materials [3]. However, there is a research gap in examining whether EFL learners’ attention could be affected by smartphone addiction while concurrently including and examining sleep quality and loneliness. Such a study could assist in the development of strategies to increase attention in EFL class and subsequently facilitate EFL learning.

University students are increasingly engaged in the usage of smartphones in their daily lives [4]. Their propensity to constantly turn to their smartphones to socialize, send brief messages, and access information has created a generation that is continually self-distracted. It has become imperative to establish a theoretical and empirical linkage between smartphone addiction and other potentially relevant variables. Such a study could help achieve desirable EFL educational outcomes, as well as equip EFL learners and educators with skills and knowledge to manage potential distractions that might come with an addiction to smartphones. 

In addition, when using smartphones in EFL class for non-educational purposes, there is a likely risk of attention being divided and students becoming disengaged with the class content. The temptation that smartphones offer may outweigh their motivation to pay attention to the EFL learning task at hand. Therefore, there is a critical need for a study to address the challenges that smartphones have brought to the EFL classroom, so that relevant stakeholders can establish optimal strategies to include smartphone technology in an EFL class with minimal downsides. 

### 1.2. Literature Review

#### 1.2.1. Smartphone Addiction

Smartphone addiction can be defined as smartphones being overused to the point of losing self-control and causing the user’s everyday life to be disturbed [5]. The addicted user feels compelled to keep using a smartphone, despite potential detriment. Because of the overuse of smartphones, the user might experience a decline in attention and memory, disturbances during sleep, eating behavior changes, and physical abnormalities, among other issues [6,7,8,9]. Distraction caused by smartphone usage while driving could increase the risk of accidents on the road [10]. It has also been reported that smartphone addiction significantly impacted students’ academic performance [11]. Compared to internet addiction, smartphones are far more addictive, simply because their numerous apps and features have more addictive qualities [12].

When the smartphone user does not have access to the device, the person might feel anxious over its absence, a symptom also known as “nomophobia” [13]. King and her colleagues [14] demonstrated that when students were addicted to smartphone usage and did not have access to the device, they experienced intense distress and anxiety. Another sign of smartphone addiction is that a user compulsively checks their phone because of an impulse to reply to messages that they are convinced need an immediate reply [15].

#### 1.2.2. Attention in EFL Class

In foreign language acquisition, attention is acknowledged as an important cognitive function [16]. Attention is a cognitive function that allows individuals to encode and store language cues in short-term and working memory as well retrieve them out of long-term memory [16]. 

Attention is also described as awareness, noticing, understanding, and consciousness, and these terms have been used interchangeably [17]. This could be owing to their instinctive subjectivity when scholars describe such notions. Based on Carr and Curran [18], to be conscious of something, people must be paying attention to it; if people are paying attention to something, then they are consciously aware of it.

Attention levels in class can fluctuate. Several techniques can increase learners’ attention in class, such as engaging students in interesting tasks [19,20], adding novelty [21], or simply catering to students’ interests [22,23]. On the other hand, students may experience an attention lapse in class when they feel tired or exhausted, lose sleep, have low mental or physical energy, or their circadian rhythm simply changes [24]. It has been found that prolonged insomnia can cause fatigue, which in turn can deteriorate task performance, but when the task becomes easier, its effect on performance is also lessened [25,26]. Performance deterioration can also happen depending on how wakeful an individual is at the time of the day. 

Learners’ working memory in a foreign language class, how well they understand the language on the whole, as well as the likely attentional resources involved could all be affected by the target language used by the teacher [27]. Furthermore, pauses, gestures, facial expression, and the voice speaking the target language can all possibly guide learners’ attention to facilitate the learning of the language [28]. Linck and his colleagues [29] noted that the processing of a foreign or second language puts demands on the resources of working memory, which is particularly evident for speakers of low proficiency. They further suggested that various cognitive functions and mental mechanisms that are necessary for the use of a second or foreign language must be supported by executive functions. This indicates that attention is required in the process of acquiring a foreign language for language comprehension as well as informational content.

Various studies have been conducted to evaluate the impact of attention on the learning of a second or a foreign language [30,31,32,33]. The findings from the research overall indicated that attention plays an important role in second or foreign language acquisition. This speaks to the need to further investigate the role of attention in an EFL classroom and to examine how it can be impacted by an addiction to smartphones and the quality of sleep.

#### 1.2.3. Sleep Quality of University Students

University students can run a high risk of having sleep-related issues. Studies have found the presence of poor sleep quality among university students. For example, sleep disturbance and deprivation are commonly found in young people [34]. The prevalence of insufficient sleep within the university student population has been noted [35]. It has been found that approximately 60% of tertiary students sleep poorly at night [36], 27% of university students suffer from a minimum of one sleeping disorder [37], 24.3% of students experience nightmares [38], and 7.7% of university students are at a risk of insomnia [39]. In addition, morning tiredness is not uncommon in university students [35].

Poor sleep quality could severely impair college students’ academic achievement, as it may impede daytime functioning [40]. It has been found that poorer GPA scores were significantly related to irregular sleep-and-waking time and shorter duration of sleep [37,41]. Curcio et al. [42] concluded that poorer academic and neurocognitive performance, as well as poorer procedural and declarative learning, were correlated with sleep problems.

#### 1.2.4. Loneliness in College Students

The transition from senior high school to university is crucial during late adolescence and early adulthood. These are periods when young adults experience social and structural changes in their roles, assumptions, and relationships [43]. College students will start making decisions independently, becoming more autonomous and learning to assimilate into the life of adulthood [44]. A new life in college also means changes in relationships with friends and family, as well as changes of domicile [45]. Though starting a new life at university may be exciting and offer new opportunities, it can also come with challenges, such as being alone in a new place and starting life in a new environment without many or any friends, which can make a young adult susceptible to loneliness.

Loneliness can be defined as a state or a situation in which someone experiences a feeling of deficiency in social or close relationships in a quantitative or qualitative way [46]. Weiss [47] proposed two categories of loneliness. These are emotional loneliness and social loneliness. When someone experiences a deficiency in networks of social relationships, such as not being socially integrated into a group of friends and acquaintances that have common interests, social loneliness arises. Emotional loneliness occurs when there is a lack of close and intimate relationships, such as losing a partner or following a divorce. Generally, loneliness has been found to affect psychological health [48].

### 1.3. Hypothesis Development

#### 1.3.1. Smartphone Addiction and Attention in Class

The theoretical underpinning of this study is mainly based upon Cognitive Load Theory [49]. Cognitive load determines how much information the brain can attend to, hold, and process at one time. Since our cognitive capacity is limited, when students engage in another task, such as using smartphones in class on top of the learning task at hand, they must load the new task into their cognitive capacity. In other words, individuals must split attention between different tasks. This places an extraneous demand on the existing cognitive load. The leaning outcome might be compromised as a result of the cognitive incapability to handle overload.

Drawing from Cognitive Load Theory, previous researchers have documented that smartphones cause distraction or affect attention during lectures in several ways. For example, McCoy [50] pointed out that the most common distractions for digital device usage during lectures for non-class-related purposes are visual and audio distractions. Campbell [51] observed that a phone ringing in the middle of class interrupted students’ attention. Froese and his colleagues [52] reported a 27% drop in test scores in students who were busy texting others in a mock classroom. Wood and her associates [53] indicated that Facebook posting, emailing, or texting had a negative impact on students’ examination performance. It has also been found that students whose attention was split between multiple tasks could easily miss significant details, and accordingly demonstrate poor school achievement [54]. In a similar study, Kuznekoff and Titsworth [55] noted that when students used mobile phones in class, they tended to write down and remember less information. Accordingly, this study hypothesizes that smartphone addiction could have a negative effect on students’ attention in EFL class (Hypothesis 1).

#### 1.3.2. Smartphone Addiction and Sleep Quality

Compared to older adults, university students need more sleep to function optimally [56]. Despite the importance of good-quality sleep for university students, it has been noted that sleeping problems found in college students are more than two times higher than in the general public [57]. Several factors, including social pressure, class starting early in the morning, demands of school work, etc. could all severely affect university students’ sleep. Another factor may be the use of smartphones before bedtime [58]. Approximately 75% of young adults mentioned that they sleep poorly as a consequence of using smartphones before bed [59]. It has been reported that university students habitually check their smartphones during awakenings at night, get woken up by incoming messages or postings on social media, or place their smartphones next to their beds at night [60]. Though various sleep problems are prevalent in young adults, less is understood about the dynamics between sleep quality and the addiction to smartphones in university EFL learners. According to the findings of previous literature, this research hypothesizes that smartphone addiction could have a negative effect on college EFL learners’ sleep quality (Hypothesis 2).

#### 1.3.3. Quality of Sleep and Attention in EFL Class

The quality of sleep has been discovered to be associated with academic performance. In a study that analyzed variables impacting the academic performance of undergraduate students, sleep management was recognized by students as a vital contributory factor to their performance [61]. The number of hours slept before exams has been identified as a factor that could explain students’ examination scores [62]. Further, a reduction in academic performance was found when there a was deterioration in the quality of sleep [63]. The influence of sleep disorders on psychomotor and cognitive performance has been documented [64,65]. A meta-analysis of 24 research projects found that adults suffering from insomnia had greater degrees of cognitive impairment in memory, in contrast to adults without a diagnosis of insomnia [65]. Additionally, students’ psychomotor performance, specifically their judgement ability, has shown a significant decrease following sleep deprivation for 20 h [64]. Inadequate sleep quality is related to the alteration of metabolic functions of the body [66], increased rates of failure, and sub-par academic performance [42].

Poor quality of sleep is also related to a deterioration in cognitive function and ability [67]. Poor quality of sleep causes a decline in memory capacity [68] and poor academic performance [69]. Individuals who stay awake late at night have been found to have attention-related issues [70]. Undergraduate students with poor sleep quality have been found to exhibit a deterioration in physical and mental health along with poor academic performance [69]. Research on consistent lack of sleep has discovered that insufficient amounts of sleep can reduce individuals’ visual and auditory attentional capabilities [71]. Drawing from previous evidence, it is reasonable to hypothesize that sleep quality can have an effect on university students’ attention in an EFL class (Hypothesis 3).

#### 1.3.4. Quality of Sleep as a Possible Mediator between Smartphone Addiction and Attention in EFL Class

The abovementioned literature has documented that smartphone addiction can affect sleep quality, and sleep quality may have an effect on attention during class. Concurrently, other research has suggested that smartphone addiction may also have a direct impact on attention in class. Based on previous evidence, this study hypothesizes that sleep quality can mediate the relationship between smartphone addiction and attention in EFL class (Hypothesis 4).

#### 1.3.5. Loneliness and Smartphone Addiction

People feel lonely when there is not enough social interaction with others [72]. Caplan et al. [73] found that lonely and socially isolated people feel a sense of relief when participating in the world of virtual reality. Ceyhan and Ceyhan [74] used three variables (computer self-efficacy, depression, and loneliness) to predict problematic internet use, and they discovered that loneliness was the most significant predictor of all. Similarly, Bozoglan et al. [75] affirmed that loneliness was the most powerful predictive factor of internet addiction among life satisfaction, self-esteem, and loneliness. In addition, Lemmens et al. [76] directly tested the relationship between loneliness and online game addiction, and they argued that online games could involve multiple players and provide an interactive social opportunity for lonely individuals. In other words, to meet their social needs, lonely individuals are more likely to be addicted to online games [73]. Therefore, this study hypothesizes that loneliness is associated with university students’ smartphone addiction (Hypothesis 5).

### 1.4. Conceptual Framework

**Figure 1:**
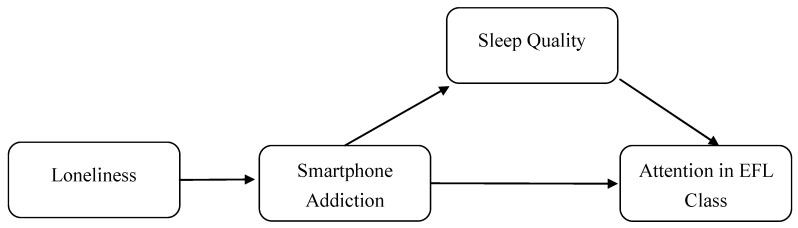
Conceptual framework.

Based on the hypotheses, this study aims to investigate a conceptual linear model that could reveal the relevant path effects of four variables: loneliness, smartphone addiction, sleep quality, and attention in EFL class (see Figure 1). This cross-sectional design within the university EFL classroom could provide quantifiable proof on the dynamics of these four variables.

## 2. Methods

This research used a non-experimental study design to gather quantitative data, in order to achieve the research goals by testing the hypotheses. This research is cross-sectional by nature. College students enrolled in EFL courses were invited to respond to survey questions. The author used self-report measures to evaluate the variables under study.

### 2.1. Participants

A total of 587 undergraduate students in Taiwan (53.49% male and 46.51% female) enrolled in EFL courses participated in the current study. The sample comprised five age groups: 18 (25.72%), 19 (44.12%), 20 (19.42%), 21 (5.28%), and 22 years old or above (5.45%). 

### 2.2. Procedure

Before the students participated in the survey, a script regarding this study and a description of the process of the survey were conveyed to them. Consent forms were given to students to inform them of the goals of this research project and to invite them to participate. If they agreed to participate, they were told that they had to answer the questionnaires intuitively and honestly; their personal answers would not have any influence on the scores of their courses, and the results of the present study were meant for research purposes only and would be kept strictly confidential. Finally, they could quit answering the survey and leave at any time. The survey data were gathered in the autumn semester of 2022. The data were anonymous. It took the students approximately 20 min to answer all questions in the survey. This study strictly abided by the guidelines of the university’s ethics committee.

### 2.3. Instruments

The short version of the Smartphone Addiction Scale (SPAS) was employed to measure the degree of severity for participants’ addiction to smartphones [77]. This instrument has 10 items on a 6-point Likert scale, ranging from 1 (strongly disagree) to 6 (strongly agree). A higher score signifies a higher risk of smartphone addiction. A sample item is: “I constantly check my smartphone so as not to miss conversations between other people on Twitter or Facebook.” This questionnaire has been found to possess robust reliability (Cronbach’s alpha = 0.91) [77].

The modified 19-item Attention in EFL Class Scale (AEFLCS) was utilized to assess attention in EFL classes [78]. This scale uses a 6-point Likert scale (1 = strongly disagree to 6 = strongly agree). A higher score indicates a higher attentional level in class. Participants were told to answer each question according to their experience in their EFL classes. A sample item reads: ”I know what the important points are in class.” This scale has been found to have internal reliability coefficients ranging from 0.83 to 0.87 and a test–retest reliability of 0.98 [78].

To measure students’ sleep quality, Urponen, Partinen, Vuori, and Hasan’s [79] Sleep Quality Index (SQI) was used. This instrument is comprised of 8 items assessing general difficulties of sleep on a 3-point Likert scale ranging from 0 (three to seven days per week) to 1 (less than three days per week) to 2 (did not happen). A higher score indicates better quality of sleep. A sample item is “Nocturnal awakenings during the past 3 months.” This questionnaire has been found to have an adequate internal reliability (Cronbach’s alpha = 0.74) [79].

The revised University of California Los Angeles Loneliness Scale (UCLALS) [80] was administered to evaluate the level of loneliness. This scale has been previously used to evaluate university students’ subjective loneliness experience [81,82]. In addition, it has also been used previously on Taiwanese university students [83]; therefore, it was considered culturally adaptable to the targeted population. This measurement has 20 items on a 4-point Likert-type scale, ranging from 1 (never) to 4 (often). A higher score on the UCLALS means a higher degree of loneliness. A sample item reads: “I feel left out.” This questionnaire has been found to possess a superb internal consistency (Cronbach’s alpha = 0.994) [84].

### 2.4. Data Analysis and Results

Collected quantitative data were analyzed with the AMOS 24 statistical software to carry out a CFA (confirmatory factor analysis) and SEM (structural equation modeling). The CFA is a prerequisite for the conceptual structural model [85]. Namely, the path effect coefficients can only be assessed after the measurement requirements are satisfied. Hair, Anderson, Tatham, and Black [86] noted that all latent variables ought to be validated using CFA before testing a structural model. The reliability of the adopted measurements was analyzed using CFA. The conceptual model of the present study was tested using SEM.

The researcher first checked the assumption of normality and discovered that it was fulfilled. The skewness and kurtosis values were within the range of ±1 and ±2 [87,88]. There was no problem of multicollinearity. All values of the variance inflation factor (VIF) were under 3 [87]. VIF is considered for ruling out the common method bias if it is less than 3.3 [89]. To show the central tendency and variability of the responses, mean (M) and standard deviation (SD), along with the correlations among the study variables, are reported (see Table 1).

### 2.5. Assessment of Measurement Model

**Figure 2:**
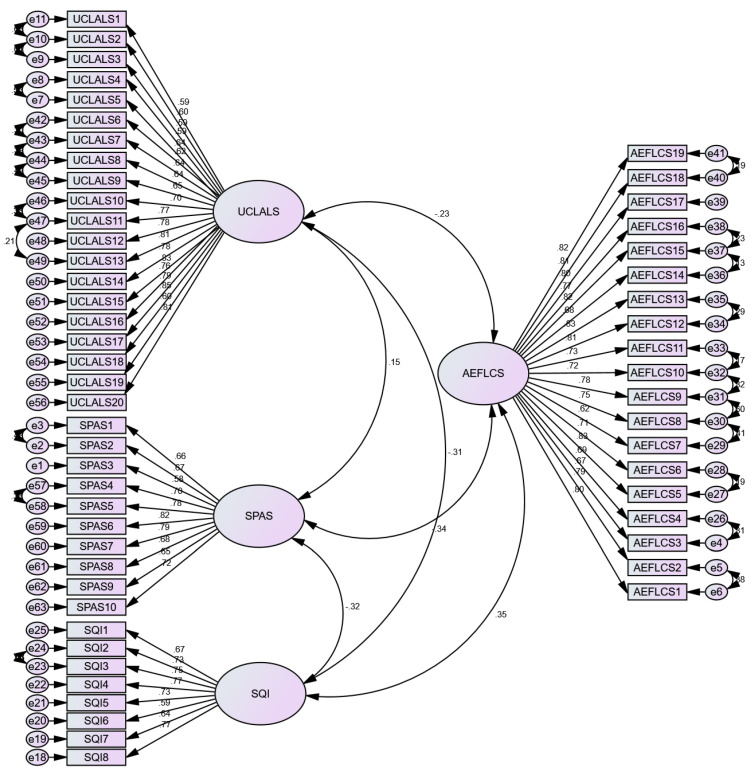
Measurement model diagram. Note: UCLALS = the revised University of California Los Angeles Loneliness Scale; SPAS = the Smartphone Addiction Scale; SQI = Sleep Quality Index; AEFLCS = the Attention in EFL Class Scale.

Confirmatory factor analysis (CFA) was carried out to analyze the dimensionality of the study scales. CFA is also a way to determine the harmony of the factor structure with the study sample and collected data [87]. Model specification was performed in line with the recommendations of Byrne [90], including re-specification to covariate the error terms to improve the model fit, which followed the criteria of modification index value > 4 [87] (see Figure 2). Subsequently, the four-factor model, which included ULCALS, SPAS, SQI, and AEFLCS, showed an acceptable fit (X^2^(3830)/df(1485) = 2.58, CFI = 0.908, TLI = 0.901, RMSEA = 0.052, RMR = 0.058) against the criteria, i.e., the X^2^/df ratio should be 3:1, CFI > 0.9, TLI > 0.9, RMSEA and RMR <0.08 [87].

The researcher also ensured that other measures on the reliability and validity of the measurement model fulfilled the required criteria. None of the factor loadings was less than 0.5 (see Figure 2), Cronbach’s alpha (CA) was >0.7, composite reliability (CR) was >0.7, convergent validity/average variance extracted (AVE) was >0.5, and for discriminant validity, the researcher checked the HTMT ratios, which were <0.85 for all study scales [87] (see Table 2).

### 2.6. Assessment of Structural Model

**Figure 3:**
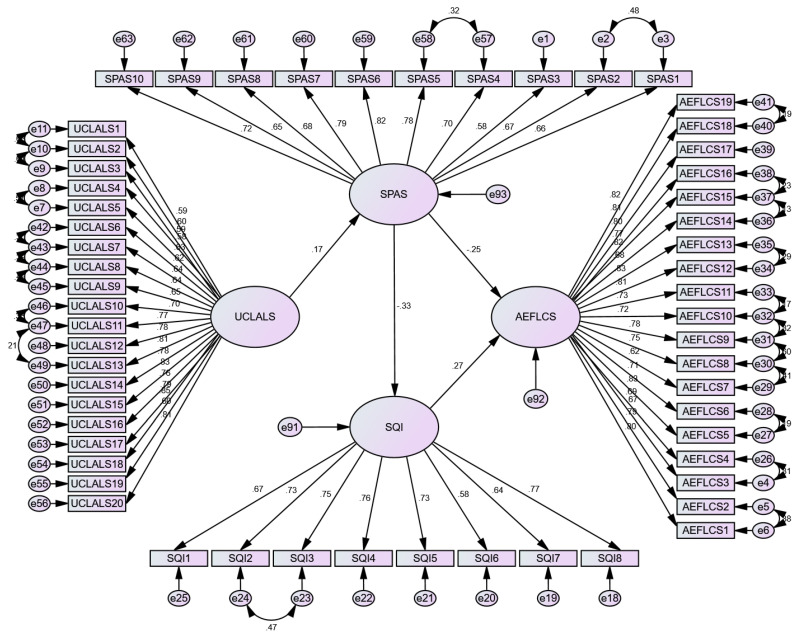
SEM path model. Note: UCLALS = the revised University of California Los Angeles Loneliness Scale; SPAS = the Smartphone Addiction Scale; SQI = Sleep Quality Index; AEFLCS = the Attention in EFL Class Scale.

Once the reliability and the validity of the measurement were verified, the researcher proceeded to test the study hypotheses, for which the researcher first checked the fit of the structural model [87]. Similar to the CFA, the author utilized AMOS 24 to assess the fitness indices of the structural model, as drawn in Figure 3. An acceptable fit was found on the four-factor structural model with the collected data (X^2^(3877)/df(1487) = 2.61, CFI = 0.906, TLI = 0.899, RMSEA = 0.052, RMR = 0.079) against the criteria, i.e., the X^2^/df ratio should be 3:1, CFI > 0.9, TLI > 0.9, RMSEA and RMR < 0.08 [87].

### 2.7. Hypothesis Testing

The researcher utilized the bias-corrected bootstrap method at 95% confidence intervals, i.e., lower-level confidence interval (LLCI) and upper-level confidence interval (ULCI). As shown in Table 3, SPAS had a negative and significant impact on AEFLCS (B = −0.252, *p* = 0.015, 95% CI (−0.333, −0.130)), which means that H1 was supported. It was concluded that smartphone addiction had a significant negative impact on attention in EFL class. SPAS also had a negative and significant impact on SQI (B = −0.326, *p* = 0.003, 95% CI (−0.423, −0.229)); therefore, H2 was also supported. It was concluded that smartphone addiction had a significant negative impact on sleep quality. Table 3 also shows that SQI had a significant and positive impact on AEFLCS (B = 0.271, *p* = 0.007, 95% CI (0.154, 0.383)), which provided obvious support to H3. It was thus concluded that sleep quality had a significant positive impact on attention in EFL class. The author noted that SQI was also able to reduce the negative impact of SPAS on AEFLCS (B = −0.094, *p* = 0.002, 95% CI (−0.180, −0.057)), meaning that there was a partial mediating effect of SQI between SPAS and AEFLCS; thus, H4 was also supported. It was concluded that sleep quality partially mediated the relationship between smartphone addiction and attention in EFL class. Lastly, H5 was also supported, as UCLALS had a positive and statistically significant impact on SPAS (B = 0.165, *p* = 0.019, 95% CI (0.044, 0.270)). It was therefore concluded that loneliness had a significant positive effect on smartphone addiction.

Although H4 was supported, the author checked the significance of the direct effect to determine the level of mediation. Since the direct effect (Table 4) was significant (B = −0.270, *p* = 0.016, 95% CI (−0.359, −0.123)), this points to the partial mediating role of SQI [91]. The original negative impact of SPAS on AEFLCS (B = −0.270) was reduced (B = −0.094) upon the introduction of SQI into the equation, meaning that only a 25.82% negative impact of SPAS on AEFLCS was carried through SQI. In other words, SQI reduced that negative impact by 74.18%. Sobel’s [92] test was conducted to further confirm this mediation effect. This test showed that there was an indirect effect of SPAS on AEFLCS via SQI (z = 4.35, *p* < 0.001); hence, H4 was further supported.

## 3. Discussion

The statistical results indicate that loneliness can lead to smartphone addiction, and smartphone addiction had a significant effect on attention in EFL class; meanwhile, sleep quality partially mediated this path. There has been a paucity of available knowledge regarding the interrelationships of these four variables. This study is an initial attempt to fill this void. Prior research has focused on each of the relationships between these four variables. This study is believed to be one of the very first to investigate their interrelationships simultaneously. Furthermore, this study offers significant and meaningful evidence with a theoretical foundation and practical implications to enhance the understanding of the four variables in the research framework.

The author found that smartphone addiction can cause poor sleep quality, and poor sleep quality subsequently impaired students’ attention in EFL class. The findings reveal that sleep quality partially accounted for the relationship between smartphone addiction and attention in EFL class. Therefore, for college students who are addicted to a smartphone, their chance to become more attentive in class could be hindered if they do not sleep well. On the contrary, those who are less addicted to a smartphone may be able to sleep better. Having better sleep quality may subsequently boost EFL learners’ attention in class, which in turn is helpful to EFL learning.

The analyses revealed that smartphone addiction had a negative effect on the sleep quality of university EFL learners. This finding supports earlier studies that have also shown that several sleep problems—including frequent awakenings at night, shorter duration of sleep, decreased night sleepiness, or insomnia—have been linked to the use of smartphones at night [93,94]. Considering that university students are often characterized by the habitual usage of smartphones at night or placing their phones near them when they sleep [60], the results of this study suggest that university students may be at risk of poor sleep quality if they use a smartphone excessively or uncontrollably.

The statistical results also showed that smartphone addiction could cause attentional lapses in EFL class. This finding correlates with previous studies [11,15], which reported that people who are addicted to a smartphone could not help but check their phone continuously. It is not uncommon to see students send text messages, play smartphone games, or post material on social media during EFL class. As a result, their minds drift from the class content, and they cannot pay attention to the teacher’s instructions. Such distraction caused by smartphones may make it difficult for students to follow instructions and complete tasks in EFL class.

The results also show that respondents demonstrated higher attention levels in EFL classes when they received a good-quality sleep. The researcher’s rationale is that better sleep quality may contribute to enhancement in cognitive function and ability [67]. High academic performance and optimum memory retention become more achievable when individuals sleep better [68,69]. Similar findings have been reported in earlier studies. For instance, it has been noted that those who have better sleep quality had less attentional problems [70]. On the other hand, students’ judgement ability was found to be significantly impaired after 20 hours of sleep deprivation [64]. It is worth mentioning that sleep deprivation and disturbances are common among young people [34]. A lack of sleep has been shown to diminish learners’ ability to pay attention to visual and auditory stimuli [71]. It is possible that sleep quality plays a role in college EFL learners’ attention in class, because it affects their psychomotor and cognitive performance.

The analyses also indicated that lonely students were prone to smartphone addiction. This finding supports previous studies and reconfirms loneliness to be one of the most significant variables in predicting smartphone addiction. Given the possibility that lonely individuals often feel inadequate in their interactions with others, either qualitatively or quantitatively, or experience deficits in close friendships or meaningful social networks, a virtual world through a smartphone provides lonely students with interactive opportunities to satisfy their social and psychological needs. Namely, in order to satisfy a desire for social relationships—close or otherwise—lonely individuals turn to a virtual world through their smartphones.

## 4. Conclusions

Until recently, the information regarding the dynamics of university students’ loneliness, smartphone addiction, sleep quality, and attention in EFL class has been scarce. Due to this dearth of research, the results of this study can expand the body of information related to this population segment. Furthermore, the findings of this research provide several noteworthy implications and important contributions for EFL education practitioners engaging with smartphone technology. Firstly, this research examined and validated a mediation model comprising the four variables. Secondly, the possible mediating effect of sleep quality might have been unnoticed by previous researchers; as a result, a bold leap of conceptual assumption was made from smartphone addiction to attention in EFL class. The present study calls attention to the need to examine potential mediators such as the quality of sleep while investigating the relationship between smartphone addiction and attention in EFL class. Thirdly, considering the direct and indirect effect of the variables, EFL instructors engaging with smartphone technology can discuss this subject with students and assist them in becoming more aware of how these factors affect their attention in EFL class. Fourthly, considering the effect of loneliness on smartphone addiction, educators can encourage students to establish real-world connections outside of the virtual world provided by the smartphone.

Given the cross-sectional nature of the data and the research design, it may not be possible to determine the direction of causality with certainty, especially considering the interrelation among the measures of variables. Therefore, drawing causal inferences should be treated with caution and is not recommended.

Considering the predictor of loneliness may play a role in smartphone addiction, and considering the mediator may have a role in students’ attention in EFL class, school counselors and mental health professionals may provide information, resources, or interventions to help college students with loneliness and poor sleep quality.

For studies in the future, researchers may consider encompassing other potentially important factors in the equation, such as foreign language anxiety or motivation, with the aim of attaining a more thorough awareness of the phenomenon of smartphone addiction and EFL learners’ attention in class.

On a final note, the present study examined these four variables carefully and analyzed the structure model statistically. The author hopes that the findings contribute to the literature on mobile technology in educational and applied linguistics settings by taking an in-depth look at college students’ loneliness, smartphone addiction, sleep quality, and attention in EFL class. As better understanding and more thorough knowledge about the dynamics of the four variables is acquired, university EFL learners can be in a better position to study and learn; instructors of EFL courses can better assist them with their achievements and goals.

## Figures and Tables

**Table 1:** Descriptive analysis.

	M	SD	UCLALS	SPAS	SQI	AEFLCS	Skewness	Kurtosis	VIF
UCLALS	2.14	0.61	-				0.120	−0.157	1.138
SPAS	3.35	1.01	0.148 *	-			0.171	−0.015	1.160
SQI	1.36	0.42	−0.313 *	−0.278 *	-		−0.754	0.487	1.242
AEFLCS	4.61	0.92	−0.248 *	−0.323 *	0.335 *	-	−0.890	1.810	1.231

Note: UCLALS = the revised University of California Los Angeles Loneliness Scale; SPAS = the Smartphone Addiction Scale; SQI = Sleep Quality Index; AEFLCS = the Attention in EFL Class Scale; M = mean; SD = standard deviation; VIF = variance inflation factor. N = 587, * *p* < 0.01.

**Table 2:** Reliability and validity analysis.

Scale	CA	CR	AVE	HTMT			
				UCLALS	SPAS	SQI	AEFLCS
UCLALS	0.957	0.952	0.295	-			
SPAS	0.911	0.909	0.507	0.158	-		
SQI	0.891	0.888	0.323	0.341	0.316	-	
AEFLCS	0.964	0.963	0.354	0.262	0.345	0.365	-

Note: UCLALS = the revised University of California Los Angeles Loneliness Scale; SPAS = the Smartphone Addiction Scale; SQI = Sleep Quality Index; AEFLCS = the Attention in EFL Class Scale; CA = Cronbach’s alpha; CR = composite reliability; AVE = average variance extracted; HTMT = heterotrait–monotrait ratio.

**Table 3:** Hypotheses testing.

Path	Estimate	LLCI	ULCI	*p*	Status
SPAS → AEFLCS	−0.252	−0.333	−0.130	0.015	H1: Supported
SPAS → SQI	−0.326	−0.423	−0.229	0.003	H2: Supported
SQI → AEFLCS	0.271	0.154	0.383	0.007	H3: Supported
SPAS → SQI → AEFLCS	−0.094	−0.180	−0.057	0.002	H4: Partially supported
UCLALS → SPAS	0.165	0.044	0.270	0.019	H5: Supported

Note: UCLALS = the revised University of California Los Angeles Loneliness Scale; SPAS = the Smartphone Addiction Scale; SQI = Sleep Quality Index; AEFLCS = the Attention in EFL Class Scale; LLCI = lower-level confidence interval; ULCI = upper-level confidence interval.

**Table 4:** Mediation analysis.

SPAS → SQI → AEFLCS	Estimate	LLCI	ULCI	*p*	Status
Indirect effect	−0.094	−0.180	−0.057	0.002	Partial mediation
Direct effect	−0.270	−0.359	−0.123	0.016	
Total effect	−0.364	−0.479	−0.231	0.009	

Note: SPAS = the Smartphone Addiction Scale; SQI = Sleep Quality Index; AEFLCS = the Attention in EFL Class Scale; LLCI = lower-level confidence interval; ULCI = upper-level confidence interval.

## Data Availability

Data is available from the author upon reasonable request.
